# Proteomic profiling of cell line-derived extracellular vesicles to identify candidate circulatory markers for detection of gallbladder cancer

**DOI:** 10.3389/fonc.2022.1027914

**Published:** 2022-11-23

**Authors:** Ratna Priya, Vaishali Jain, Javed Akhtar, Neeraj Saklani, Puja Sakhuja, Anil Kumar Agarwal, Ravindra Varma Polisetty, Ravi Sirdeshmukh, Sudeshna Kar, Poonam Gautam

**Affiliations:** ^1^ Laboratory of Molecular Oncology, Indian Council of Medical Research (ICMR)- National Institute of Pathology, New Delhi, India; ^2^ Jamia Hamdard- Institute of Molecular Medicine, Jamia Hamdard, New Delhi, India; ^3^ Department (NIL), Academy of Higher Education (MAHE), Manipal, India; ^4^ Department of Pathology, Govind Ballabh Pant Institute of Postgraduate Medical Education and Research (GIPMER), New Delhi, India; ^5^ Department of GI Surgery, Govind Ballabh Pant Institute of Postgraduate Medical Education and Research (GIPMER), New Delhi, India; ^6^ Department of Biochemistry, Sri Venkateswara College, University of Delhi, New Delhi, India; ^7^ Institute of Bioinformatics, International Tech Park, Bangalore, India

**Keywords:** gallbladder cancer (GBC), NOZ, OCUG-1, extracellular vesicles, proteomics, haptoglobin

## Abstract

Gallbladder cancer (GBC) is the sixth most common gastrointestinal tract cancer with a very low overall survival and poor prognosis. Profiling of cancer-derived extracellular vesicles (EVs) is an emerging strategy for identification of candidate biomarkers for the detection and prognosis of the disease. The aim of the study was to analyse the protein content from GBC cell line- derived EVs with emphasis on proteins which could be used as candidate biomarkers for the detection of GBC. NOZ and OCUG-1 cell lines were cultured and EVs were isolated from conditioned media. LC-MS/MS analysis of total EV proteins led to the identification of a total of 268 proteins in both the cell lines. Of these, 110 proteins were identified with ≥2 unique peptides with ≥2 PSMs in at least two experimental and technical replicate runs. STRING (Search Tool for the Retrieval of Interacting Genes/Proteins) database was used to perform bioinformatics analysis of 110 proteins which showed ‘cell adhesion molecule binding’, ‘integrin binding’, ‘cadherin binding’ among the top molecular functions and ‘focal adhesion’ to be among the top pathways associated with the EV proteins. A total of 42 proteins including haptoglobin (HP), pyruvate kinase (PKM), annexin A2 (ANXA2), thrombospondin 1 (THBS1), were reported to be differentially abundant in GBC tissue. Of these, 16 proteins were reported to be differentially abundant in plasma and plasma-derived EVs. We infer these proteins to be highly important to be considered as potential circulatory biomarkers for the detection of GBC. To check the validity of this hypothesis, one of the proteins, haptoglobin (HP) as a representative case, was analysed in plasma by quantitative Enzyme- linked immunosorbent assay (ELISA) and we observed its increased levels in GBC in comparison to controls (p value= 0.0063). Receiver operating characteristic (ROC) curve analysis for GBC vs controls showed an Area under the ROC Curve (AUC) of 0.8264 for HP with 22% sensitivity against 100% specificity. We propose that HP along with other candidate proteins may be further explored for their clinical application.

## Introduction

Gallbladder cancer (GBC) is the lethal disease of gallbladder and ranked as sixth most common gastrointestinal (GI) tract cancer. It is more prevalent in Asian countries which contribute 70% of worldwide new GBC cancer cases. According to GLOBOCAN 2020 database the estimated number of new GBC cases worldwide in 2020 is 115949 and within India the estimated new cases are 19570 (1). The disease is usually detected at the advanced stages due to vague symptoms and lack of highly specific and sensitive panel of biomarkers for the detection which results in a very low overall survival and poor prognosis (2). The current diagnostic methods used for the detection of GBC include ultrasound, computed tomography (CT) scans, magnetic resonance imaging (MRI) and biochemical tests including liver function tests (LFT) and cancer antigens (CEA and CA19-9) (3, 4). Cancer antigens (CA) have been widely utilized as diagnostic markers for GBC and other GI tract tumors, however, the sensitivity and specificity of these antigens is low for GBC which limits their application as reliable biomarkers in the diagnosis of GBC (2).

EVs are small heterogeneous vesicles and their contents depend on the type of cellular source, state and environment (5). Profiling of cancer-derived extracellular vesicles is an emerging strategy for the identification of diagnostic and prognostic markers for various cancers. Various groups have analyzed EVs from cancer cell lines for identification of protein signatures specific to a particular cancer cell line which may be useful for diagnosis and prognosis of cancer. Hurwitz et al. has profiled the EV proteome of a total of sixty cell lines (NCI 60) which led to the identification of 6071 proteins. Overall, they found 1500 proteins to be differentially expressed in EV samples derived from 60 cell lines (representing signature proteins of each cell lines), which may directly lead to the discovery of biomarkers of cancer, ultimately affecting diagnosis and prognosis of the increasingly prevalent disease (6). One of the groups studied and compared the proteome of EVs derived from human primary colorectal cancer cells (SW480) and their metastatic derivatives (SW620) which resulted in understanding of the role of EVs in the metastasis and led to identification of potential biomarkers for cancer metastasis (7). A study conducted by Guerreiro et al. has compared the protein content of EVs derived from three different cancer cell lines- pancreatic ductal adenocarcinoma (PDAC), Oral squamous cell carcinoma (OSCC) and melanoma brain metastasis cell lines which led to identification of an EV specific candidate biomarkers characteristic of each cancer which could be further studied and analysed for diagnosis and prognosis of the disease (8). In line with this, we analyzed protein content carried by EVs derived from two GBC cell lines, NOZ and OCUG-1, which could be potential diagnostic biomarker for detection of GBC. This will lead to identification of candidate markers which may be explored to develop a highly sensitive and specific test for the detection of GBC.

## Methodology

### GBC cell lines

The human gallbladder cancer cell lines (NOZ, OCUG-1) were obtained from Japanese Collection of Research Bioresources (JCRB) cell bank, Japan (9).

### Cell culture

NOZ cells were cultured in Williams’ E medium supplemented with 10% fetal bovine serum (FBS) and OCUG-1 cells were cultured in Dulbecco’s modified Eagle’s medium (DMEM), 0.5mM pyruvate, 2mM glutamine and 10% FBS at 37 °C in humidified air with 5% CO2. After cell lines reached 75-80% confluency, NOZ and OCUG-1 cells were washed with Phosphate-buffered saline (PBS) and cultured in Williams’ E medium and DMEM respectively with Exo-free FBS (conditioned medium) for 24 h. The cells were collected for cell viability assay or protein extraction and the conditioned medium was used for EV isolation.

### Cell viability assay

Cell viability assay was performed using trypan blue method (10). Briefly, the cells were collected and resuspended in serum free medium. One part of resuspended cells is mixed with equal volume of 0.4% trypan blue dye, allowed to incubate for 2 min and the cell count was done by using hemacytometer under light microscope.

### EV isolation and characterization

#### EV isolation

The EVs were isolated using ultracentrifugation based method (11). Briefly, the culture medium was collected and centrifuged at 300 × g for 10 min to remove the cells. The supernatant was collected and centrifuged at 16,500 × g for 30 min at 4°C to remove cell debris followed by filteration through 0.22 µm filters. After filtration, the media was centrifuged at 1,10,000 × g for 2 h at 4°C. The EV pellet was then washed with PBS and centrifuged at 1,10,000 × g for 2 h at 4°C. The supernatant was discarded and the pellet containing EVs were resuspended in PBS and RIPA buffer [25 mM Tris-Cl, pH 7.6 + 150 mM NaCl + 2% 3-{(3-cholamidopropyl) dimethylammonio-1-propanesulfonate (CHAPS)} with 0.5% protease inhibitor cocktail (Sigma, USA)] for characterization and total protein isolation respectively.

#### Nanoparticle tracking analysis

EVs resuspended in PBS were analyzed for size and concentration by NTA using a NanoSight LM20 system (Malvern, UK) as described earlier (12). Samples were introduced manually and the video images were recorded for 60 s using the NTA software (version 3.1) with camera level- 16 and screen gain- 10. Processing of images was performed with detection threshold 3 and screen gain 10. Each video was analyzed to obtain the mode vesicle size and the concentration. For all the samples, NTA acquisition settings were kept constant. Each experiment was carried out in duplicates. The NanoSight was calibrated with 20 nm, 60 nm and 120 nm latex beads.

#### Transmission electron microscopy

EVs resuspended in PBS was loaded on carbon-coated grids. The sample was washed with MQ water twice followed by negative staining performed using 2% phosphotungstic acid (PTA). Images of EVs were acquired using TEM (200KV, TECNAI G20 HR-TEM, Thermo Scientific) at 1,04,000**×** magnification.

#### Protein extraction and SDS-PAGE analysis

EV pellet was dissolved in modified RIPA buffer followed by sonication (Biologics 3000MP, USA) with four bursts of 10 s each with 10 s of pause interval at 4°C for protein extraction. Total EV protein was estimated by Bradford assay (13). The whole Cell lysates from both the cell lines were prepared by resuspending the cells in RIPA buffer followed by sonication as described previously. A total of 15 µg protein from cell lysate and EV protein from both the GBC cell lines was loaded on Sodium dodecyl-sulfate polyacrylamide gel electrophoresis (SDS-PAGE) as described earlier by Priya et al. (12). Coomassie Brilliant Blue R250 was used to stain the gel and the Image was acquired using imaging system (ChemidocMP, Bio-Rad, USA).

### Protein identification and quantitation

#### iTRAQ labeling

Cell line-derived EV proteins from NOZ and OCUG-1 (20 µg each group) were subjected to trypsin digestion and the peptides were labelled with iTRAQ reagents according to the manufacturer’s instructions (iTRAQ Reagents Multiplex kit; Applied Biosystems/MDS Sciex, CA, USA) and as described earlier by Sahasrabuddhe et al. (14). iTRAQ labelling was used for the advantage of multiplexing. The EV protein digest from two experimental replicates of NOZ was labelled with 114 and 115 whereas, EV protein digest from two experimental replicates of OCUG-1 was labelled with 116 and 117 tags respectively. All the four labelled peptide samples were pooled, vacuum-dried and subjected to strong cation exchange (SCX) cartridge for clean-up followed by desalting using C18 cartridge (Pierce, Rockford, USA) as per the manufacturer’s instructions for further LC-MS/MS analysis. iTRAQ experiment was performed in triplicates. The flow chart with the experimental design for identification of EV proteins in the two GBC cell lines is shown in **Supplementary Figure S1**.

#### LC-MS/MS analysis

Nanoflow electrospray ionization tandem mass spectrometric analysis was carried out using QExactive plus (Thermo Scientific, Bremen, Germany) interfaced with Dinonex RS nanoLC 3000 nanoflow LC system. The desalted Peptides from each exp[erimental replicates were enriched using a C18 trap column (75 μm × 2 cm) at a flow rate of 3 μl/min and fractionated on an analytical column (75 μm × 50 cm) at a flow rate of 300 nl/min using a linear gradient of 8–35% acetonitrile (ACN) over 85 min. Mass spectrometric analysis was performed in a data dependent manner using the Orbitrap mass analyzer at a mass resolution of 70,000 at m/z 200. For each MS cycle, 10 top most intense precursor ions were selected and subjected to MS/MS fragmentation and detected at a mass resolution of 35,000 at m/z 200. The fragmentation was carried out using higher-energy collision dissociation (HCD) mode. Normalized collision energy (CE) of 30% was used to obtain release of reporter ions from all peptides detected in the full scan. The ions selected for fragmentation were excluded for next 30 sec. The automatic gain control for full FT MS and FT MS/MS was set to 3e6 ions and 1e5 ions respectively with a maximum time of accumulation of 50 msec for MS and 75 msec for MS/MS. The lock mass with 10 ppm error window option was enabled for accurate mass measurements (12). Three replicate LC-MS/MS runs were performed.

### Data analysis

Protein identification, quantification and annotations of proteins were carried out as described earlier (12). The MS/MS data was analyzed using Proteome Discoverer (Thermo Fisher Scientific, version 2) with Mascot and Sequest HT search engine nodes using NCBI RefSeq database (release 81). Search parameters included trypsin as the enzyme with 1 missed cleavage allowed; precursor and fragment mass tolerance were set to 10 ppm and 0.1 Da, respectively; Methionine oxidation and deamidation of asparagines and glutamine was set as a dynamic modification while methylthio modification at cysteine and iTRAQ modification at N-terminus of the peptide and lysines were set as static modifications. The peptide and protein information were extracted using high peptide confidence and top one peptide rank filters. The FDR was calculated using percolator node in proteome discoverer 2.0. High confidence peptide identifications were obtained by setting a target FDR threshold of 1% at the peptide level. Proteins identified in at least two experimental and technical replicates and in both the cell lines with ≥2 unique peptides were considered significant and used for further analysis. The proteins with ≥ 1.5 fold change (OCUG-1 vs NOZ) were used to identify proteins enriched in each cell line.

### Bioinformatics analysis

Mapping of proteins identified in both the cell lines and in at least two replicates with ≥2 unique peptides for associated molecular functions and pathways was done using the STRING (Search Tool for the Retrieval of Interacting Genes/Proteins) database (https://string-db.org/) (15). The localization of the proteins was identified using UniProt database (www.uniprot.org) (16).

### Clinical samples

Blood samples from adult patients diagnosed with GBC, GSD (gallstone disease) and healthy individuals were collected from Govind Ballabh Pant Institute of Postgraduate Medical Education and Research (GIPMER), New Delhi after approval from Institutional Human Ethics Committee. GBC cases with ≥20 year age group and adenocarcinomas were included for the study. GBC cases with age <20 years or having malignancy other than GBC or those who have already taken the treatment were excluded for the study. Tumor Staging was done on the basis of clinical data of patients, histopathological evaluation and imaging tools, as per American Joint Committee on Cancer (AJCC), 8^th^ edition staging system. The control group did not have any malignancy. ‘Healthy volunteers’ or ‘GSD cases with no detectable dysplasia’ with ≥20 year age group were included in control group. The control group did not have any malignancy.

Peripheral blood was collected from patients with early stage GBC (Stage I and II, n=6), advanced stage GBC (stage III and IV, n= 12), GSD cases (n=7) before surgery and from healthy individuals (n=9). The samples were processed within 30 min of collection for the separation of plasma. The samples were centrifuged at 2000 × g for 20 min at 4°C, clear plasma separated, aliquoted and stored at -80°C for ELISA. **Table 1** shows details of the clinico-pathological features of the samples used in the study.

**Table 1:** Clinical samples used in the study.

Subjects	Total number	Number of males	Number of females	Mean age (Years)	Age range (Years)
**Total GBC Cases**	**18**	**2**	**16**	**49.22**	**34-66**
Histological Grade					
Well-differentiated (G1)	6	2	4	47.66	38-60
Moderately-differentiated (G2)	7	0	7	47.28	34-65
Poorly-differentiated (G3)	5	0	5	53.8	50-66
Gallstone*					
Present	9	2	7	51.44	34-66
Absent	4	0	4	52	42-53
**Total Controls**	**16**	**2**	**14**	**43.12**	**25-62**
GSD cases	7	1	6	46.28	37-62
Healthy group	9	1	8	40.66	25-53

*No gallstone information was available for 5 GBC cases. The Bold values highlight the total numbers in each category.

### Enzyme- linked immunosorbent assay

Plasma level of human haptoglobin (HP) was measured in individual plasma samples from cases (GBC cases, n= 18) and controls (healthy individuals, n=9 and GSD cases, n=7) using ELISA quantitation kit (Abcam, USA). Concentration of Haptoglobin is presented as scatter plot and statistical analysis was performed using GraphPad Prism 5 (www.graphpad.com) (17). Differences in protein levels between two independent groups was tested with Unpaired t-test (two-tailed) with confidence intervals of 95% and *p*-value less than 0.05 was used to indicate statistical significance as described earlier by Priya et al. (12). Receiver operating curve (ROC) analysis for HP for GBC vs all controls was performed leading to the estimates of area under the curve (AUC) with 95% confidence interval (CI) along with sensitivity and specificity.

## Results

**Figure 1:**
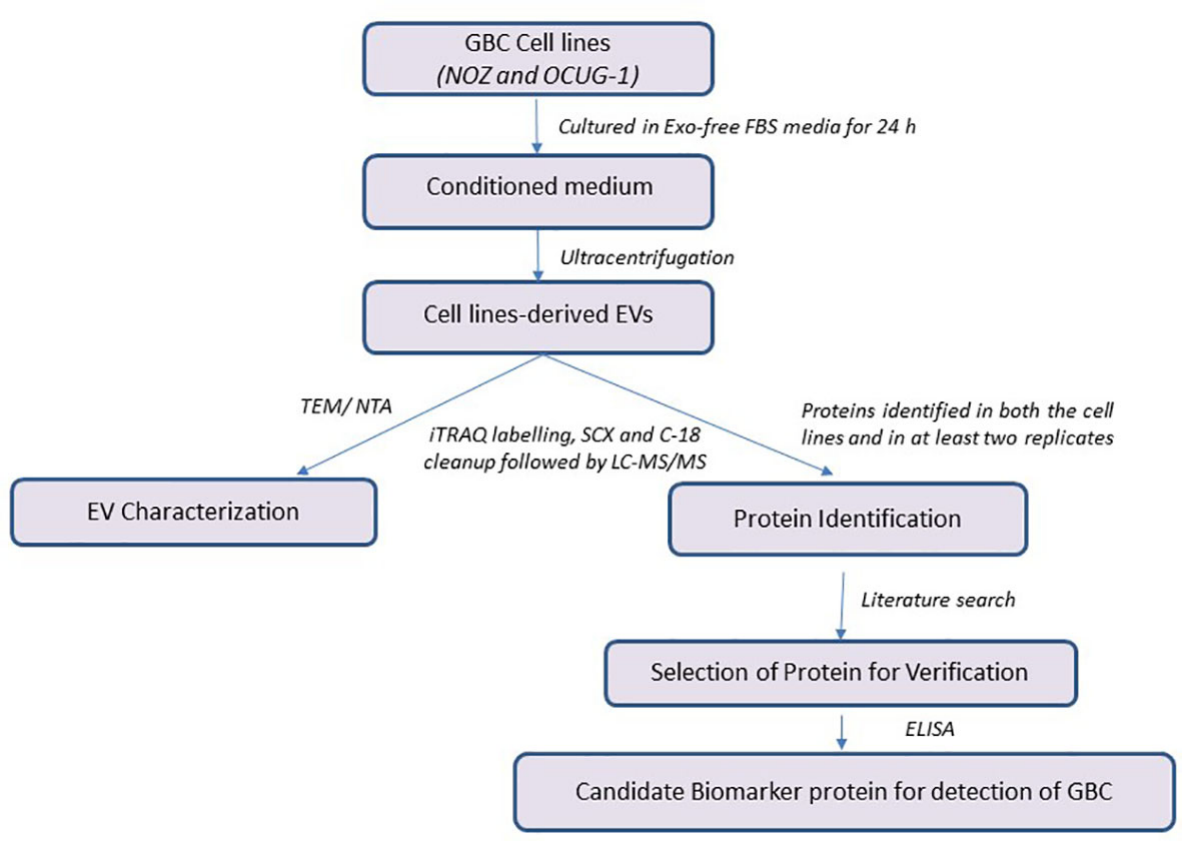
Workflow of the study. GBC, Gallbladder carcinoma; EV, Extracellular Vesicles; iTRAQ, Isobaric tags for relative and absolute quantitation; ELISA, Enzyme-linked immunosorbent assay.

In the present study, we have analyzed EV proteome from two GBC cell lines, NOZ and OCUG-1 followed by bioinformatics analysis to understand their relevance in terms of molecular functions and pathways. Further, one of the proteins was verified in blood plasma from GBC cases and controls (healthy volunteers, GSD cases). The study design is shown in **Figure 1**.

### EV characterization

**Figure 2:**
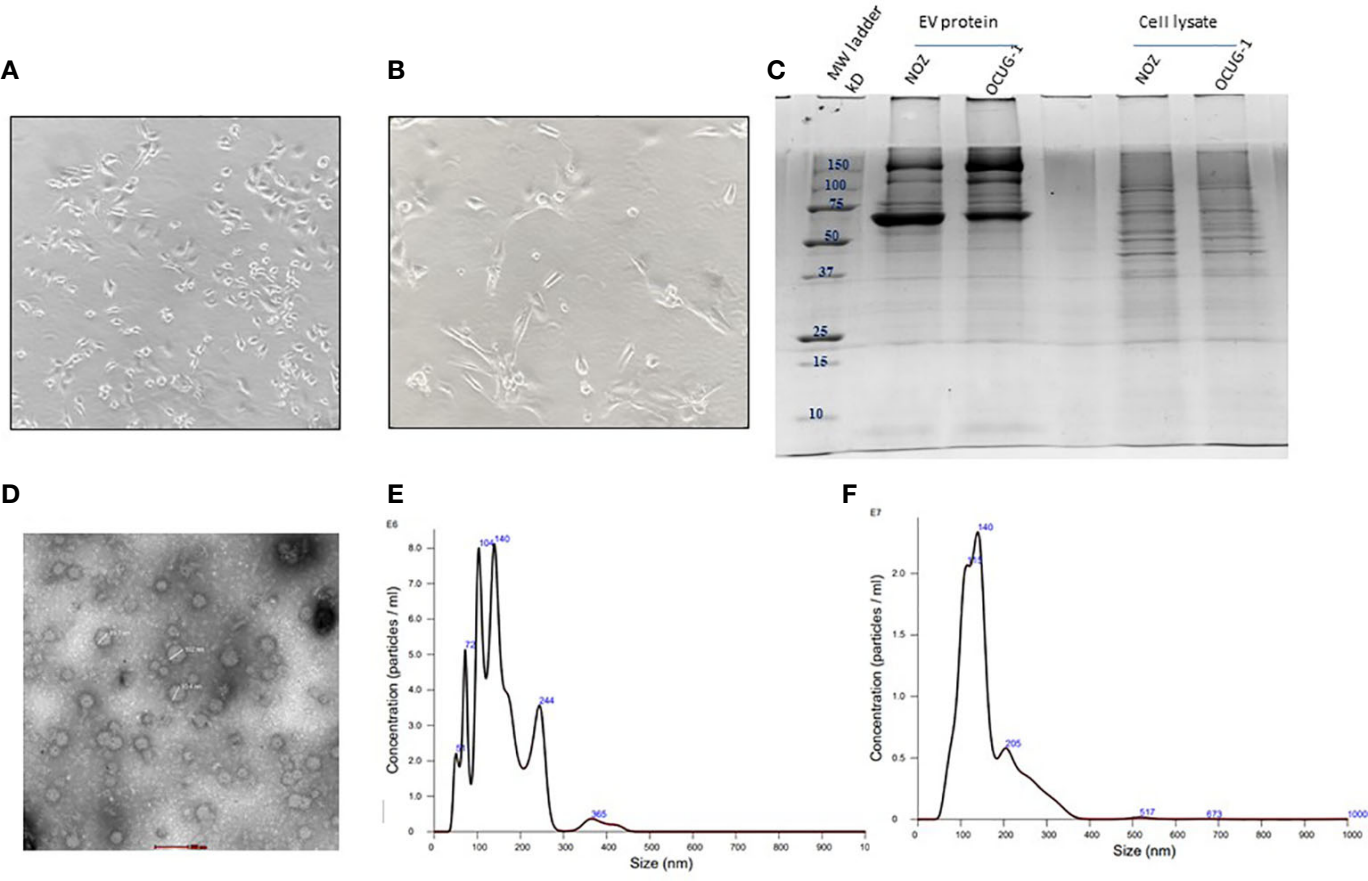
Characterization of GBC cell line-derived EVs. Representative image of GBC cell line NOZ **(A)** and OCUG-1 **(B)**. SDS-PAGE profile of GBC cell line-derived EV proteins and cell lysate **(C)**. Transmission electron micrographs of cell line- derived EVs. EVs from cell lines were isolated by ultracentrifugation method were resuspended in PBS and loaded on 2% carbon coated grids. Negative staining was performed using 2% phosphotungstic acid (PTA). Images of EVs (40-100 nm) were acquired using 200KV, TECNAI G20 HR-TEM, Thermo Scientific at 1,00,000× magnification, scale bar- 100 nm **(D)** Size and particle distribution plots of EVs from GBC cell line-derived EVs using nanoparticle tracking system showed peaks at 140 nm and 139 nm for NOZ **(E)** and OCUG-1 **(F)** respectively suggesting enrichment of exosomes in the EV fraction.

Cell viability assay performed after 24 h of replenishing with Exo-free FBS media showed ≥95% viability for NOZ and OCUG-1 cells indicating no significant cell death at the time of collection of media for EV isolation. The cell line images are shown in **Figures 2A, B** and **Supplementary Figures S2A, S2B**. SDS-PAGE profile of EV proteins from NOZ and OCUG-1 showed different protein profile when compared with cell lysates (NOZ and OCUG-1) with enrichment of high molecular weight proteins (**Figure 2C**). TEM analysis of GBC cell line-derived EVs showed size ranging from 40-100 nm. **Figure 2D** shows the representative transmission electron micrograph of GBC cell line-derived EVs. The particle and size distribution plots of EVs by NTA analysis showed a mode of 140 nm for NOZ and 139 nm for OCUG-1, suggesting enrichment of exosomes in our EV preparation (see **Figures 2E, F**).

### Protein identification

GBC cell line-derived EV proteome analysis led to the identification of a total of 268 proteins (**Supplementary Table S1**). Of these, 110 proteins were identified in both the cell lines (**Supplementary Table S2**). Based on PSMs, we screened top 25 proteins enriched in EVs which includes talin-1 (TLN1), actin (ACTG1), pyruvate kinase (PKM), transforming growth factor-beta-induced protein (TGFB1), tubulin alpha-1B chain (TUBA1B), phosphoglycerate kinase 1 (PGK1), filamin-A isoform 2 (FLNA) along with others (**Supplementary Table S3**). Comparison between the two cell lines showed a total of 19 proteins to be enriched in NOZ cells such as lactotransferrin isoform 1 (LTF), alpha-2-HS-glycoprotein isoform 2 (AHSG), albumin (ALB), heat shock protein HSP 90-alpha isoform 1 (HSP90AA1) whereas, 10 proteins were found to be enriched in OCUG-1 cells including interstitial collagenase isoform 1 (MMP1), apolipoprotein B-100 (APOB), tubulin beta chain isoform b (TUBB) (**Supplementary Table S4**).

### Bioinformatic analysis

**Figure 3:**
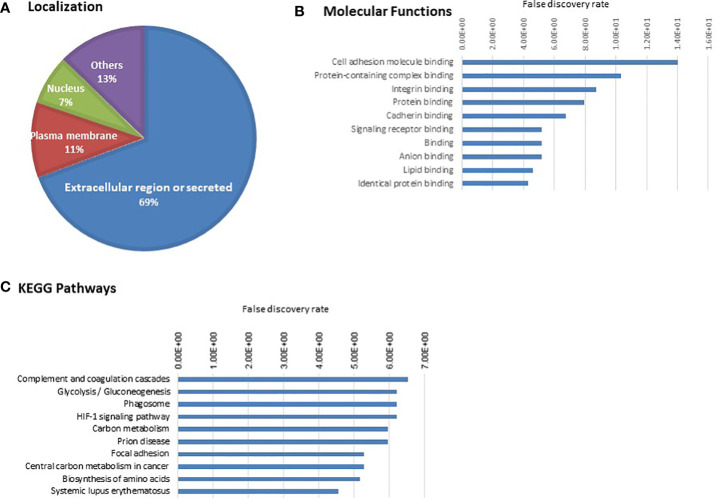
Gene ontology analysis of 110 cell line-derived EV proteins. **(A)** Localization **(B)** Molecular Function **(C)** KEGG pathways. The proteins associated with the top 10 molecular functions and pathways are provided in **Supplementary Tables S5A** and **S5B**.

Annotation of 110 proteins for localization showed 76 belonging to extracellular region, 13 in plasma membrane, 8 in nucleus (**Figure 3A**). The top molecular functions include cell adhesion molecule binding, protein-containing complex binding, integrin binding (**Figure 3B** and **Supplementary Table S5A**). Some of the important cell adhesion proteins already reported to have a role in cancer progression are included, namely, TGFB1, Thrombospondin (THBS1), integrin or related proteins [integrin-linked protein kinase (ILK), integrin beta-1 (ITGB1)], insulin-like growth factor II (IGF2), (PKM), vinculin (VCL). The top pathways include complement and coagulation cascade, glycolysis/gluconeogenesis, phagosomes, HIF-1 signalling pathway (**Figure 3C** and **Supplementary Table S5B**).

### Clinical verification by ELISA

**Figure 4:**
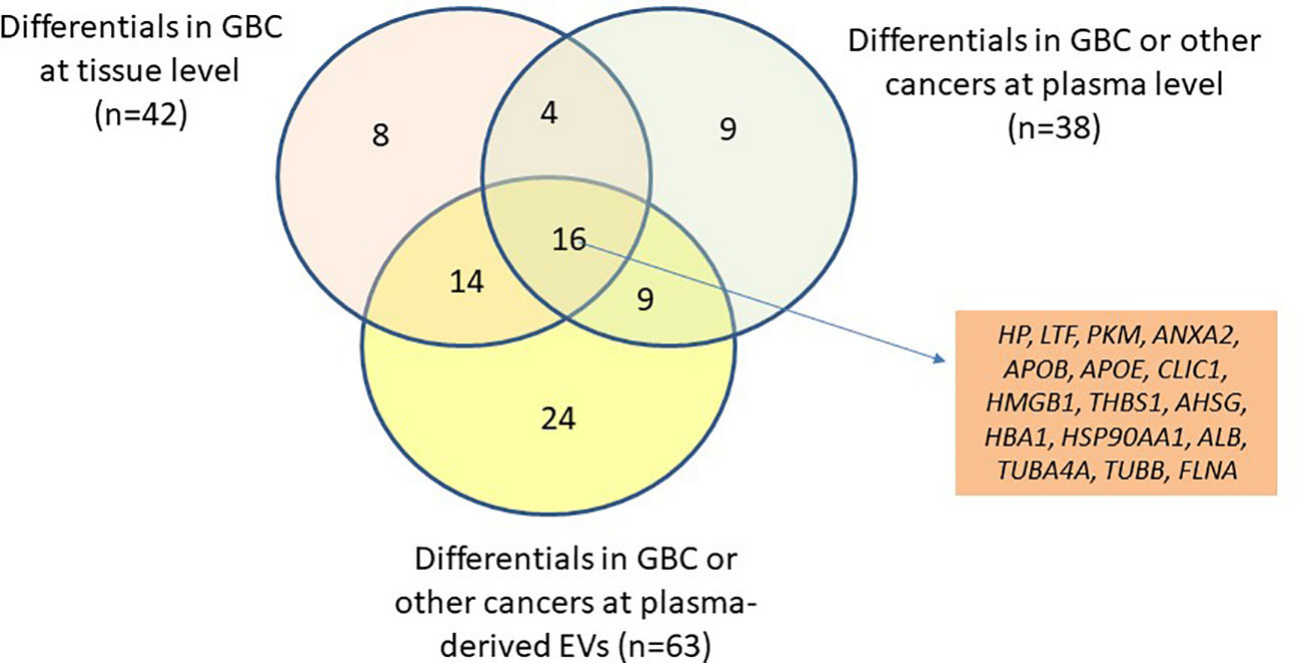
Venn diagram showing the differential level of 110 proteins in tissue (GBC) and in plasma/serum or plasma/serum-derived EVs (GBC or other cancers). The differential level of 16 proteins were already reported in tissue, plasma/serum and plasma/serum-derived EVs and are candidate proteins to be explored as circulatory markers for GBC. *HP, Haptoglobin; LTF, lactotransferrin isoform 1; PKM, pyruvate kinase isoform X1; ANXA2, annexin A2; APOB, apolipoprotein B-100; APOE, apolipoprotein E isoform a; CLIC1, chloride intracellular channel protein 1; HMGB1, high mobility group protein B1 isoform X1; THBS1, thrombospondin-1; AHSG, alpha-2-HS-glycoprotein isoform 2; HBA1, hemoglobin subunit alpha; HSP90AA1, heat shock protein HSP 90-alpha isoform 1; ALB, serum albumin; TUBA4A, tubulin alpha-4A chain isoform 1; TUBB, tubulin beta chain isoform b; FLNA, Filamin-A isoform 2*.

**Figure 5:**
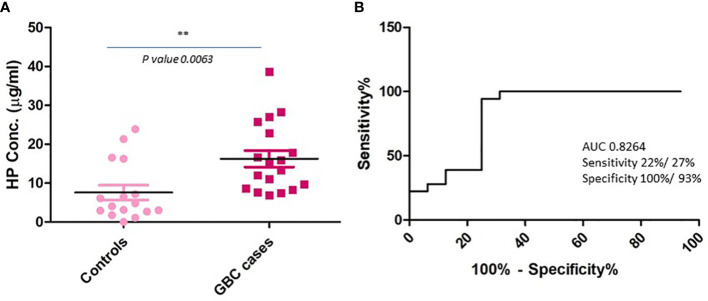
Plasma level of HP in GBC cases and controls using quantitative ELISA. **(A)** Scatter plot showing concentration in plasma samples. Controls include healthy individuals and GSD cases. A significant increase in the levels of HP was observed in GBC cases **(B)** ROC curve representing sensitivity and specificity for HP. ** p value is less than or equal to 0.01.

Based on the literature survey, 42 out of 110 proteins were found to be differentially abundant in GBC at tissue level and 76 to be differentially abundant in plasma or plasma-derived EVs from GBC various cancers including GBC, while 16 of them were common across them and included HP, PKM, ANXA2, THBS1 (**Figure 4**, **Table 2**; **Supplementary Table S2**). Plasma level of one of the proteins, haptoglobin (HP), was measured in individual plasma samples from cases (GBC n= 18) and controls (healthy individuals, n=9, GSD cases, n=7) using ELISA quantitation kit (Abcam, USA) and results are represented as scatter plot in **Figure 5A**. The mean value of HP for GBC was 16.27 ± 2.11 µg/ml, in comparison to controls i.e. healthy individuals and GSD which was 8.723 ± 2.743 µg/ml and 6.123 ± 1.894 µg/ml respectively. Comparison of HP levels in GBC cases with all controls showed a significant increase in GBC (p value= 0.0063) (**Figure 5A**). Receiver operating characteristic (ROC) curve analysis for GBC vs controls showed an Area under the ROC Curve (AUC) of 0.8264 (95% CI: 0.6078- 0.8988) for HP with 22% sensitivity against a specificity of 100% (**Figure 5B**).

**Table 2:** List of 16 proteins reported to be differentially abundant in GBC tissue as well as in plasma and plasma-derived EVs.

SNo.	Gene symbol	Protein name	Localization (Uniprot)	Molecular Functions (STRING database)
1	AHSG	alpha-2-HS-glycoprotein isoform 2	Secreted	Peptidase regulator activity, Endopeptidase inhibitor activity, Enzyme regulator activity
2	ALB	serum albumin	Extracellular region or secreted	Protein binding, Anion binding, Lipid binding, Identical protein binding
3	ANXA2	annexin A2 isoform 2	Extracellular region or Secreted	Cell adhesion molecule binding, Protein binding, Cadherin binding, Anion binding, Lipid binding
4	APOB	apolipoprotein B-100	Extracellular region or Secreted	Protein binding, Signaling receptor binding, Anion binding, Lipid binding
5	APOE	apolipoprotein E isoform a	Extracellular region or Secreted	Protein-containing complex binding, Protein binding, Signaling receptor binding, Anion binding, Lipid binding, Identical protein binding
6	CLIC1	chloride intracellular channel protein 1	Plasma membrane	Cell adhesion molecule binding, Protein binding, Cadherin binding, Binding
7	FLNA	Filamin-A isoform 2(Short name: FLN-A)	Extracellular region or secreted	Protein-containing complex binding, Protein binding, Cadherin binding, Signaling receptor binding, Identical protein binding
8	HBA1	hemoglobin subunit alpha	Extracellular region or secreted	Binding, Small molecule binding, Ion binding
9	HMGB1	high mobility group protein B1 isoform X1	Extracellular region or secreted	Cell adhesion molecule binding, Protein-containing complex binding, Integrin binding, Signaling receptor binding, Protein binding
10	HP	Haptoglobin isoform 1	Extracellular region or secreted	Protein binding, Binding
11	HSP90AA1	heat shock protein HSP 90-alpha isoform1	Plasma membrane	Protein-containing complex binding, Protein binding, Anion binding, Identical protein binding
12	LTF	lactotransferrin isoform 1 (Short name:Lactoferrin)	Extracellular region or secreted	Binding, Anion binding, Lipid binding
13	PKM	pyruvate kinase PKM isoform X1	Extracellular region or secreted	Cell adhesion molecule binding, Protein-containing complex binding, Protein binding, Cadherin binding, Anion binding, Identical protein binding
14	THBS1	thrombospondin-1	Extracellular region or Secreted	Cell adhesion molecule binding, Protein-containing complex binding, Integrin binding, Signaling receptor binding, Anion binding, Lipid binding, Identical protein binding
15	TUBA4A	tubulin alpha-4A chain isoform 1	Cytoplasm, Cytoskeleton	Protein binding, Binding, Anion binding
16	TUBB	tubulin beta chain isoform b	Cytoskeleton	Protein-containing complex binding, Protein binding, Signaling receptor binding, Anion binding

## Discussion

EVs are an important mediator of cell to cell signaling and have been associated with cancer progression and metastasis. EV proteins are being considered for diagnostic and therapeutic applications in various cancers. Here, we studied GBC cell line-derived EV proteome and identified EV proteins associated with cancer development and progression which could be useful as circulatory marker for detection of GBC. Proteomic analysis of two GBC cell lines (NOZ and OCUG-1) identified a total of 110 proteins detected in both the cell lines and in at least two experimental and technical replicates. Of these, 19 proteins were found to be enriched in NOZ cells while 10 proteins were enriched in OCUG-1 cells. Out of 110 proteins, 87 were associated with cancer cell proliferation, invasion and migration, while 69 proteins were associated with angiogenesis. ‘Cell adhesion molecule binding’ was found to be the top molecular function which includes proteins associated with TGF beta signaling pathway [TGFB1, ILK, ITGB1, THBS1, connective tissue growth factor(CTGF)] suggesting their potential role in development and progression of GBC. Based on the literature survey, 42 out of 110 proteins were found to be differentially abundant in GBC at tissue level and 76 to be differentially abundant in plasma or plasma-derived EVs in GBC or other cancers, while 16 of them were common (**Figure 4**, **Table 2**; **Supplementary Table S2**). Earlier, we identified 86 proteins to be differentially abundant in plasma-derived EVs in GBC. Here, we found a total of 21 proteins be common among the cell line-derived EVs (identified in the present study) which includes HP, cofilin-1 (CFL1), pyruvate kinase (PKM), proteasome subunit alpha type-5 (PSMA5) (11). One of the functionally relevant proteins, haptoglobin (HP), was analyzed by quantitative ELISA and found increased levels in GBC in comparison to controls.

Bioinformatic analysis of 110 proteins showed ‘cell adhesion molecule binding’, ‘integrin binding’, ‘cadherin binding’ among the top molecular functions and ‘focal adhesion’ to be among the top pathways associated with the EV proteins. The role of cell adhesion proteins in cancer cells is well reported (18). Primary tumor-derived EVs are reported to stimulate epithelial cells to activate epithelial-mesenchymal transition (EMT) process resulting in the loss of tumor cell adhesion and release of tumor cells into the circulation leading to the spread of tumor cells to distant sites (19). Interestingly, the ‘cell adhesion molecule binding’ group includes proteins associated with ‘TGFB1 signaling’ which is reported to promote EMT and invasion in advanced stages of cancer (20). These proteins include TGFB1, THBS1, integrin or related proteins (ILK, ITGB1), CTGF and all of them are earlier reported to be overexpressed in GBC tissue (21–24). THBS1, a multi-functional matricellular ECM and secreted protein, is reported to activate the latent TGFB1 homodimers resulting in TGFB1 signaling (25). TGFB1 overexpression in GBC has been correlated with advanced stage and poor patient survival and reported to promote cancer cell proliferation migration and invasion (21, 26). TGF-β1 is reported to induce CTGF expression and promote metastasis of gastric cancer (27). CTGF is also reported to have a pro-growth activity in gallbladder cancer cells (24). Serreno et al. reported the role of ILK activity in TGFB1-inducted EMT in breast cancer (28). ITGB1 is reported to regulate TGF-beta 1-mediated p38MAPK activation and EMT progression (29).

The functional role of EV-derived TGFB1 and ILK in cancer has been reported earlier. TGFB1 is also reported to be present in EV in multiple cancers (30). TGFB1 containing EVs has been reported to bind to the receptors present on the recipient cells and activating SMAD dependent or SMAD independent signaling regulating the expression of oncogenes (PI3K, AKT, N-cadherin, vitronectin, MMPs) (30) and promoting tumor cell proliferation, invasion. ILK-expressing EVs derived from primary tumor are reported to promote EV uptake in the recipient cells which may further promote activation of cancer-associated signaling in the cells (30). Overall, we found TGF beta signaling associated proteins in GBC-derived EVs, however, their functional role in tumor progression and development needs to be established in GBC.

We performed literature search in order to screen EV proteins which have the potential to be explored in blood plasma or plasma-derived EVs for detection of GBC. We found 16 proteins including HP, PKM, ANXA2, THBS1 that are differentially abundant in GBC tissue (literature search and unpublished data from our lab), in plasma and plasma-derived EVs from GBC or other cancer patients (**Table 2**). Earlier, we reported differential abundance of 86 proteins in plasma-derived EVs from GBC cases (12). Comparison of 110 proteins with them showed 21 proteins to be common, of these, 7 proteins [HP, Proteasome subunit alpha type-5 (PSMA5), Proteasome subunit beta type-1 (PSMB1), Cofilin 1 (CFL1), apolipoprotein B-100 (APOB), histone H2B type 1-D isoform X1 (HIST1H2BD), ILK] were found to have increased levels in GBC.

One of the functionally relevant proteins, haptoglobin, was further selected for clinical verification using individual plasma samples. Haptoglobin is an acute phase protein which is mainly synthesized in liver and is reported to be overexpressed in various types of cancers including GBC and reported to promote cell proliferation, migration and invasion, which implies its role in pathophysiological process of GBC (31–33). Clinical verification of HP by quantitative ELISA in the present study showed significantly increased levels in GBC in comparison to controls (health volunteers and GSD cases) (**Figure 5A**). ROC curve analysis showed 22% sensitivity against a specificity of 100% (**Figure 5B**). In our previous study, we found increased level (1.7 fold) of HP in plasma-derived EVs in advanced stage GBC (12). Another study by Tan et al. showed an increased level of serum HP in GBC cases by Western blot analysis, however the sample size was limited (32). We find our data to be in correlation with the previously reported study, however the plasma HP may be analyzed in large cohort of samples.

## Conclusions

The present study analyzed GBC cell line-derived EV proteome and identified 110 proteins in two GBC cell lines with high confidence. The proteins associated with top molecular function ‘Cell adhesion’ includes ‘TGF beta signaling’ related proteins which are reported to be involved in EMT. Based on the literature search, we screened 16 proteins as potential circulatory markers and verified one of the proteins, HP, which showed increased plasma levels in GBC patients. We believe that HP alongwith remaining other proteins in combination may be further explored for their potential as circulatory markers for detection of GBC.

## Data availability statement

The original contributions presented in the study are included in the article/**Supplementary Material**. Further inquiries can be directed to the corresponding authors.

## Ethics statement

The studies involving human participants were reviewed and approved by Clinical samples from participants visiting GIPMER, Delhi, were collected for the study after approval from the Institutional Human Ethics Committee [MAMC-IEC (No: F.1/IEC/MAMC (51/5/2015/No 12) and NIP-IEC/21-12/04)]. All the participants provided informed consent to participate in the study and written informed consent was obtained. The patients/participants provided their written informed consent to participate in this study.

## Author contributions

PG, RS, and SK designed the experiment. RP, VJ, and JA did data acquisition. PS, AA, RP, VJ, JA, and NS contributed to clinical sample collection and clinical data management. Analysis and interpretation of data was done by PG, RVP, RP, NS, VJ, PS, and SK. Drafting and editing of the manuscript was done by PG, RP, PS, AA, RS, RVP, and SK. All authors contributed to the article and approved the submitted version.

## Funding

The work reported here was financially supported by Indian Council of Medical Research (ICMR) (Project ID- 2014-1182 and 2020-0109), Govt. of India, New Delhi. RP is a Ph.D. student registered at Jamia Hamdard, New Delhi and DST- Innovation in Science Pursuit for Inspired Research (INSPIRE)- Senior Research Fellow (SRF). VJ is a Ph.D. student registered at recipient of Senior Research Fellowship (SRF) from the Council of Scientific and Industrial Research (CSIR), Govt. of India. JA is a Ph.D. student registered at Jamia Hamdard, New Delhi and a recipient of Senior Research Fellowship (SRF) from the ICMR- National Institute of Pathology, Govt. of India. NS is Junior Research Fellow (JRF) under ICMR project (Project ID-2020-0109).

## Acknowledgments

We acknowledge Dr. Seishi Nagamori, Department of Virology II, National Institute of Infectious Diseases, Tokyo, Japan, who established NOZ cell line. We acknowledge Dr. Amit Kumar Dinda, Professor, All India Institute of Medical Sciences (AIIMS), New Delhi, India for NTA analysis to characterize EVs. We are thankful to Sophisticated Analytical Instrument Facility (SAIF), AIIMS, New Delhi, India, for TEM analysis to characterize EVs. We acknowledge Dr. Ankit P. Jain from Institute of bioinformatics, Bangalore, India for LC-MS/MS analysis.

## Conflict of interest

The authors declare that the research was conducted in the absence of any commercial or financial relationships that could be construed as a potential conflict of interest.

## Publisher’s note

All claims expressed in this article are solely those of the authors and do not necessarily represent those of their affiliated organizations, or those of the publisher, the editors and the reviewers. Any product that may be evaluated in this article, or claim that may be made by its manufacturer, is not guaranteed or endorsed by the publisher.

## Abbreviations

GBC, Gallbladder carcinoma; EVs, Extracellular vesicles; GSD: Gallstone disease; iTRAQ, isobaric tags for relative and absolute quantitation; ELISA, Enzyme-linked immunosorbent assay; ROC, Receiver operating characteristic curve; AUC, Area under the ROC Curve; NTA, Nanoparticle tracking analysis; SCX, Strong cation exchange; LC-MS, Liquid chromatography–mass spectrometry; FDR, False discovery rate; PSMs, Peptide spectral matches; STRING, Search Tool for the Retrieval of Interacting Genes/Proteins.
