# Supplementary material for: Proteomic profiling of cell line-derived extracellular vesicles to identify candidate circulatory markers for detection of gallbladder cancer
Source: Front Oncol. 2022 Nov 23;12:1027914. doi: 10.3389/fonc.2022.1027914 (PMC9727277; doi:10.3389/fonc.2022.1027914)
Supplement: Supplementary file 2 [file Presentation_1.pptx]

## Slide 1
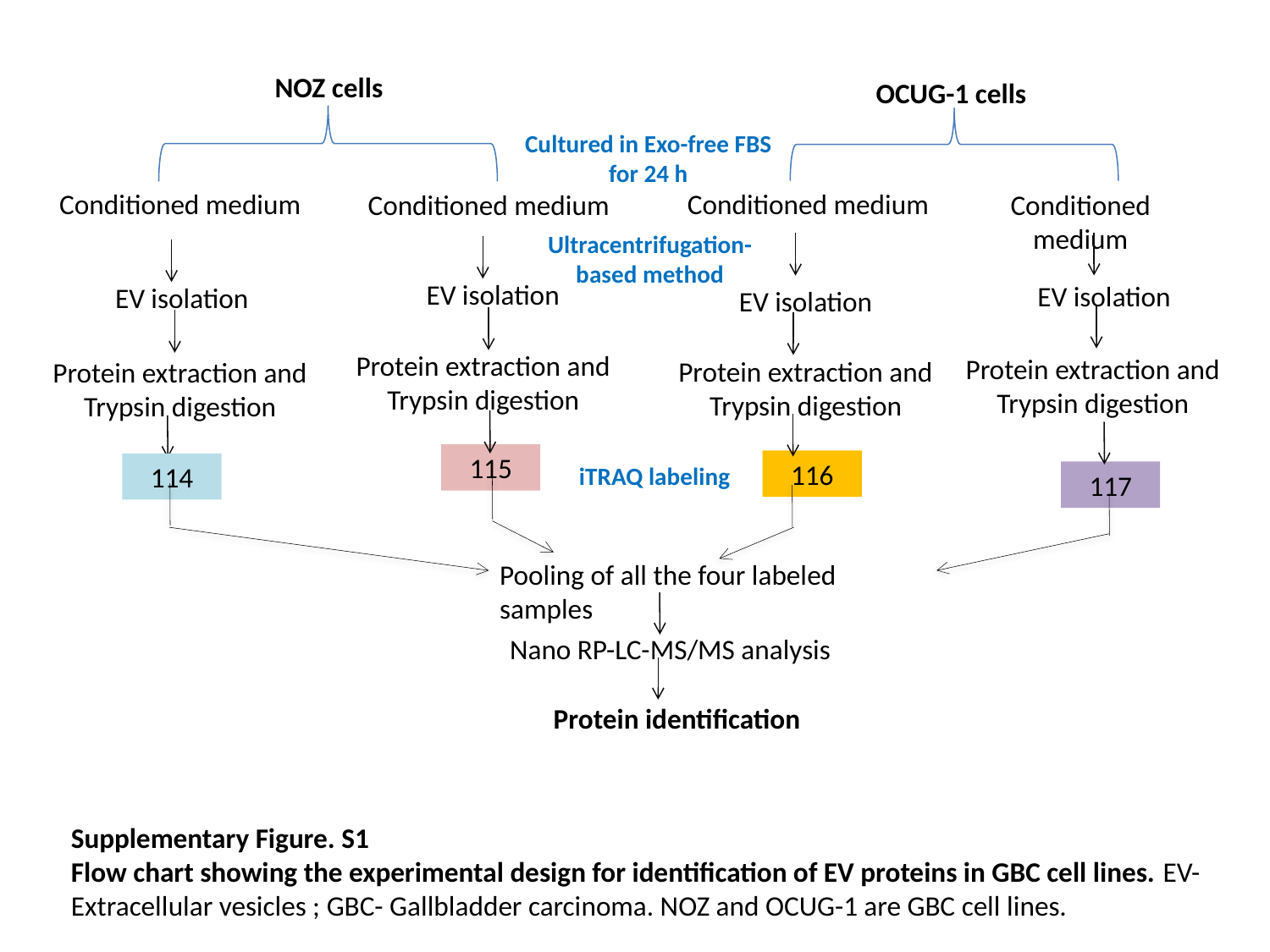

NOZ cells
OCUG-1 cells
Conditioned medium
Conditioned medium
Conditioned medium
Conditioned medium
Ultracentrifugation-based method
EV isolation
EV isolation
EV isolation
EV isolation
Protein extraction and Trypsin digestion
Protein extraction and Trypsin digestion
Protein extraction and Trypsin digestion
Protein extraction and Trypsin digestion
115
116
114
iTRAQ labeling
117
Pooling of all the four labeled samples
Nano RP-LC-MS/MS analysis
Protein identification
Cultured in Exo-free FBS for 24 h
Supplementary Figure. S1
Flow chart showing the experimental design for identification of EV proteins in GBC cell lines. EV- Extracellular vesicles ; GBC- Gallbladder carcinoma. NOZ and OCUG-1 are GBC cell lines.

## Slide 2
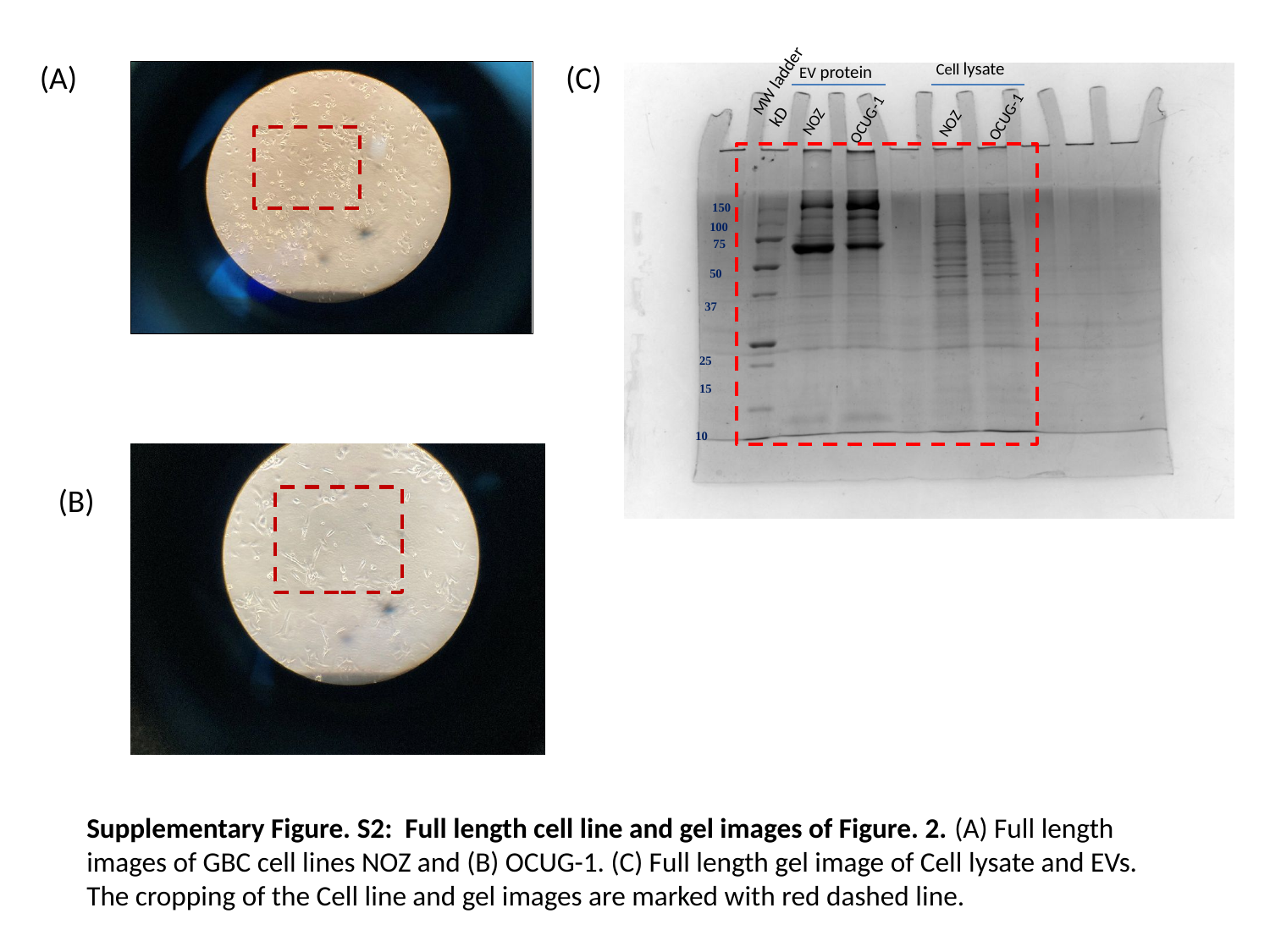

(A)
(C)
Cell lysate
EV protein
MW ladder
kD
OCUG-1
OCUG-1
NOZ
NOZ
150
100
75
50
37
25
15
10
(B)
Supplementary Figure. S2: Full length cell line and gel images of Figure. 2. (A) Full length images of GBC cell lines NOZ and (B) OCUG-1. (C) Full length gel image of Cell lysate and EVs. The cropping of the Cell line and gel images are marked with red dashed line.
